# Hypothesis: Potentially Systemic Impacts of Elevated CO_2_ on the Human Proteome and Health

**DOI:** 10.3389/fpubh.2020.543322

**Published:** 2020-11-16

**Authors:** Carlos M. Duarte, Łukasz Jaremko, Mariusz Jaremko

**Affiliations:** ^1^Red Sea Research Centre and Computational Bioscience Research Center, King Abdullah University of Science and Technology, Thuwal, Saudi Arabia; ^2^Bioscience and Environmental Science and Technology Division, King Abdullah University of Science and Technology, Thuwal, Saudi Arabia

**Keywords:** CO_2_, pH, climate-change, human, health

## Abstract

Uniform CO_2_ during human evolution (180 to 280 ppm) resulted, because of the role of the CO_2_-bicarbonate buffer in regulating pH, in rather constant pH (7.35 to 7.45) in human fluids, cells and tissues, determining, in turn, the narrow pH range for optimal functioning of the human proteome. Herein, we hypothesize that chronic exposure to elevated *p*CO_2_ with increasing atmospheric CO_2_ (>400 ppm), and extended time spent in confined, crowded indoor atmospheres (*p*CO_2_ up to 5,000 ppm) with urban lifestyles, may be an important, largely overlooked driver of change in human proteome performance. The reduced pH (downregulated from 0.1 to 0.4 units below the optimum pH) of extant humans chronically exposed to elevated CO_2_ is likely to lead to proteome malfunction. This malfunction is due to protein misfolding, aggregation, charge distribution, and altered interaction with other molecules (e.g., nucleic acids, metals, proteins, and drugs). Such alterations would have systemic effects that help explain the prevalence of syndromes (obesity, diabetes, respiratory diseases, osteoporosis, cancer, and neurological disorders) characteristic of the modern lifestyle. Chronic exposure to elevated CO_2_ poses risks to human health that are too serious to be ignored and require testing with fit-for-purpose equipment and protocols along with indoor carbon capture technologies to bring CO_2_ levels down to approach levels (180–280 ppm) under which the human proteome evolved.

## Introduction

pH is a key factor that determines the performance of the human proteome, as the chemical reactions involving proteins occur mainly in aqueous phases or at the interface between aqueous phases and biological membranes (1). pH ranges narrowly around 7.35, typically from 7.25 to 7.44, in human and mammalian blood and well-irrigated tissues (2) such as the brain (3) and the lungs (4). The narrow pH range in human blood and tissues is consistent with the optimal pH human proteome performance, which has been experimentally demonstrated to be narrowly constrained around 7.35 (Table 1). Deviations in pH from this optimal value may, thus, affect the performance of the human proteome and, more broadly, a range of additional biomolecules.

**Table 1:** Examples of different structural and physical changes resulting in the pH sensitive functioning of the human proteome and the associated health syndromes.

**Induced structural /physical properties changes**	**Protein**	**Compartment**	**Response to reduced pH**	**Observation**	**Possible/related syndrome or metabolic implications**	**References**
Protein folding/induced structural changes	Human Serum Albumin (HSA)	Blood	HSA exists in its neutral state at pH of about 7.35, and has been reported to experience functional changes as pH declines.	HSA is the most abundant redox agent in the blood by affecting the presence of reduced cysteine 34 (-SH). Reduced pH affects the activity and redox function of HSA through the protonation of histidine 39, altering the redox potential of cysteine 34.	Multiple metabolic dysfunctions connected with the transport of various biomolecules (e.g., free fatty acids or cholesterol, drugs and xenobiotics) through blood. In the case of certain medications, like warfarin, the altered affinity to albumin may lead to a free warfarin plasma level increase and consequently bleeding.	(S1–S3)
	Hemoglobin	Blood	A decrease in pH below 7.4 readily reduces the affinity of hemoglobin for O_2_	The pH-dependence of O_2_ binding to hemoglobin is a classical case of allostery, referred to as the “root effect,” leading to protonated histidine 146 that shifts deoxyhemoglobin to the T state, which has a lower affinity for oxygen (i.e., Bohr effect).	Blood acidosis, apnea, impaired intellectual abilities, and eventually, at extremely low pH compromising viability and leading to potentially lethal complications.	(S4)
	Carbonic Anhydrase (CA, e.g., CA XII)	Skeleton including cytosol, mitochondria and plasma membrane.	Activity increases at pH <7.4 to reach a maximum activity at pH ~7.00, and declining at lower pH.	Structural, allosteric changes of carbonic anhydrases are induced upon the reduction in pH (<7.0) with their activity being strongly altered, associated with progressive unfolding of the protein.	Removal of excess CO_2_ through enhanced CA activity leads to bone structures increasingly depleted in Ca^2+^, increasing the likelihood of osteroporosis, at pH <7.4, impacts may include possible cancer development (renal cancer, lung cancer). CAi activity has been tentatively suggested to act as a cancer transducer for *p*CO_2_ fluctuations (arising from intermittent blood flow), therefore adding to the role of CO_2_ as a potent cytoplasmic signal.	(S5–S10)
	Epithelial sodium channel (EnaC)	Primarily kidney nephrons, and also lung cells, testes and ovaries	As pH declines below 7.35 the ENaC gating is enhanced, leading to increased Na^+^ transport.	Reduced pH facilitates Na^+^ reabsorption across the apical membranes of epithelia in the distal nephron, respiratory and reproductive tracts, and exocrine glands. This affects water homeostasis, with the cells responding through increased polyubiquitination of β-ENaC.	These changes lead to increasing systemic ionic imbalance, affecting general homeostasis. Under acute impacts (e.g., very low pH), EnaC may interact with CFTR (Cystic Fibrosis Transmembrane conductance Regulator) possibly playing a role on cystic fibrosis.	(S5, S8, S11)
	Mechanistic target of rapamycin complex 1 (mTORC1)	Golgi Apparatus	rmTORC1, which has a central role in regulating many fundamental cell processes, from protein synthesis to autophagy, shows a reduced activity when pH declines below 7.4.	The effect of reduced pH on mTORC1 activity is a reversible process, but is yet to be characterized from a structural biology perspective.	Multiple health disorders can be observed when mTORC1 signaling is deregulated, including the progression of cancer and diabetes, as well as enhanced aging processes.	(S12, S13)
	1-antitrypsin (1-AT)	Blood	Decreasing pH leads to a decrease in the inhibitory activity of 1-antitrypsin (1-AT).	Although the pH-dependence of protein activity is well-established, the specific impacts on the molecular structure of 1-antitrypsin (1-AT) have not yet been characterized.	Changes in molecular structure of 1-antitrypsin (1-AT) have been associated with deteriorated lung and kidney function and, specifically, idiopathic respiratory distress syndrome (IRDS) in children.	(S14–S16)
Changing in binding affinity to small molecules (Ligand/drug/metal/fatty acids)	Orosomucoid (alpha1-acid glycoprotein)	Blood	pH-dependence of binding affinities of Orosomucoid toward various small organic ligands.	Reduced affinity under lowered pH has been reported for a range of tested drugs, including dipyridamole, nicardipine and imipramine.	Case of changed activity of Dipyridamole which is a medication that inhibits blood clot formation, when given chronically (or activity increases as in case of lower pH) and causes blood vessel dilation when given at high doses over a short time.	(S17)
	Prion protein	Brain	Decreasing pH has been reported to affect the binding of histidine residues in the flexible n-terminal part of the protein as well as binding of transition metal ions (e.g., Cu^2+^, Zn^2+^).	Existing evidence suggests that, with decreasing pH, the transition from soluble PrP^c^ to pathogenic insoluble PrP^sc^ accelerates. Increased aggregation of prions in pathogenic forms leads to neurodegenerative processes.	Increased aggregation of prion protein in pathogenic forms PrP^sc^ leads to neurodegenerative processes.	(S18)
Phase separation (e.g., membrane-less organelles)	Nucleoporins (NUPs) transporting proteins and RNA across the nucleus membrane; proteins and/or nucleic acids that undergo membrane-less compartmentalization	All nucleus-containing human cells	Binding and translocation of proteins by NUPs is strongly pH-dependent. Whether this applies to RNA as well is still unresolved. Although the details of pH sensitivity of human proteins undergoing liquid-liquid phase separation have yet to be studied in detail, it might be expected to at least some of them by analogy to known pH-sensitive protein systems from yeast.	Binding and translocation of proteins by NUPs is strictly regulated by protein electrostatic surface, which is strongly pH-dependent.	Negative implications on cellular organization, signaling, RNA processing and transport through cellular membranes. Cell division process perturbed resulting in symptoms typical of neurodegenerative diseases as well as a likelihood of cancer development, among many other impacts.	(S19–S26)
Protein/DNA interactions altered	RNA-binding proteins (RBPs)	All nucleus-containing human cells	Interactions of RBPs with proteins and nucleic acids have been reported to be highly pH-dependent, within the clear-cut thresholds, as they are mainly electrostatically-driven.	RBPs are prone to mis-folding and aggregation with decreasing pH. The specific mechanism, which likely involves electrostatic changes from charge redistribution, has been examined for just a few case-studies and there is still uncertainty as to its general applicability across RBPs.	pH-dependent RBP misfunction has important metabolic implications connected to neurodegenerative diseases, and possible increases of the risk for cancer development. As RNA-binding proteins' transcriptional and post-transcriptional regulation of RNA have a role in regulating the patterns of gene expression during development, their misfunction can have systemic consequences.	(S27, S28)
Aggregation, surface charge distribution changes	Amylin	Langerhans isles in the pancreas	Whereas the pH ranges studied are broader than the functional range relevant for humans, there is strong evidence for pH-dependence of amylin.	Decreasing pH below 7.35 leads to the protonation of His18 and decrease of aggregation as well as formation of channels in the membrane environment.	The main consequence is the likelihood of release and subsequent aggregation of amylin, which is linked to the possibility of developing diabetes Type II.	(S29)
	Synucleins (S) and β-amylid peptides (Abeta)	Neuronal cells	Aggregation of synucleins and β-amylid peptides (rate and/or stability of formed aggregates) has been shown to be pH-dependent.	Decreasing pH may serve as an on/off switch for βS fibrilization, and as a modulator of αS and Abeta aggregation. Moreover, pH-dependent changes in charge distribution along amino acid sequences also affect the peptides' aggregation properties.	Aggregation of synuclein and Abeta is likely conducive to neurogenerative diseases, such as Parkinson's and Alzheimer's.	(S30, S31)
	Transthyretin (TTR)	Blood	Decreasing pH leads to enhanced formation of aggregates.	With decreasing pH, soluble oligomers may be formed, eventually causing amyloid formation. Lowered pH might increase the improperly folded tetramer levels and shift the equilibrium toward less stable tetramers and monomers.	Unstable TTR tetramers and monomers enhance pathological amyloid or deposit formation.	(S32)
	Glutathione S-transferases (GSTs)	All nucleus-containing human cells	The activity of GSTs was found to be pH dependent within the range of pH of relevance.	Whereas detailed structural studies of GST with decreasing pH are lacking, the observed decline in activity is likely to involve ionization of active groups affecting the 3-D structure and dynamics of the protein.	GST is master protein accounting for as much as 10% of the proteins in the human cytosol.	(S33–S35)

*References provided in Supplementary Table 1*.

pH buffering in human blood is mainly controlled by the CO_2_-bicarbonate buffer system (1). In a reversible reaction catalyzed by carbonic anhydrase, gaseous CO_2_ reacts with H_2_O to form carbonic acid, which rapidly dissociates into bicarbonate and hydrogen ions. pH homeostasis by the CO_2_-bicarbonate buffer system is aided by additional buffering elements, including partially protonated histidine or cysteine side chains, the *N*-terminal α-amino groups of proteins, and organic/inorganic phosphate groups (5). Indeed, the marine CO_2_-bicarbonate system was preserved during the evolution of metazoans, initiated in the ocean about 700 Myrs ago (6), as the abundant buffer primarily responsible for pH homeostasis in metazoan fluids and tissue compartments (7). Indeed, CO_2_-bicarbonate is also the main buffer responsible for pH homeostasis in human fluids (2, 5, 8, 9).

**Figure 1:**
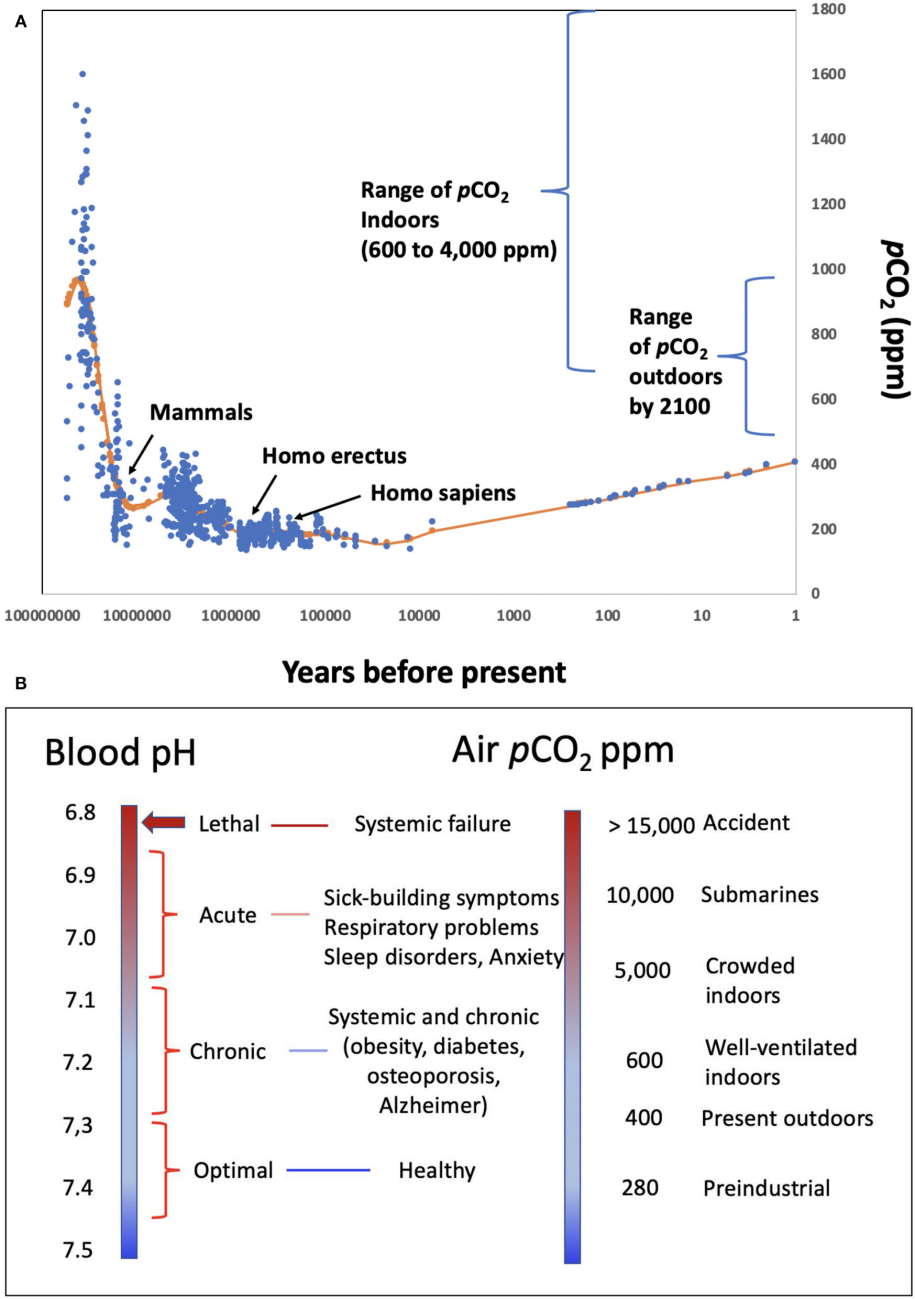
**(A)** *p*CO_2_ levels in the atmosphere experienced throughout the evolution of mammals and humans, as well as the range of *p*CO_2_ characteristic of indoors environments and the range expected by 2,100 under different emission scenario pathways (19); **(B)** correspondence between different levels of ambient *p*CO_2_, and the environments where these may be encountered, and expected blood pH values and human condition.

However, the CO_2_-bicarbonate buffering system also links the pH in animal fluids, cells, and tissues to the *p*CO_2_ (partial pressure of carbon dioxide) in the ambient atmosphere [(2, 5, 10–12), Figure 1]. This resembles the link between ocean surface pH and atmospheric CO_2_, which is also mediated by the oceanic CO_2_-bicarbonate buffering system (13). As a result, changes in *p*CO_2_ in air results in a change of the blood pH in a matter of minutes (14, 15). The control ambient CO_2_ exerts on the pH of human blood (2, 16, 17) is then transferred to the pH regime of tissues irrigated by blood, which reflect changes in ambient CO_2_ (2).

Atmospheric *p*CO_2_ has narrowly fluctuated between 135 and 280 ppm along glacial-interglacial cycles between the appearance of humans, about 1 million years, and the onset of the industrial revolution (Figure 1). During this period, the pH in human blood would have ranged narrowly between 7.35 and 7.45 for venous and arterial blood, respectively (18) (Figure 1), encompassing the optimal buffering capacity of the carbonate buffer.

The recent rise in atmospheric *p*CO_2_ that currently exceeds 400 ppm has exposed humans to atmospheric *p*CO_2_ levels unprecedented through the evolution of humans and mammals (Figure 1). This implies a slight decline of blood pH below the 7.35–7.45 optimal performance range for the human proteome, similar to the decline in ocean pH that has occurred since the industrial revolution (13, 20). The major change in human exposure to elevated *p*CO_2_, however, has arisen because of a shift in lifestyles. Until the 1950s, most people spent significant time outdoors. Now, people are living in increasingly confined and crowded spaces, such as those in heated and air-conditioned buildings and vehicles. Europeans, for example, currently spend over 90% of their time indoors [> 21 h per day; (21)]. *p*CO_2_ typically ranges from 600 to 800 ppm in well-ventilated indoor areas to 4,000 ppm or above in crowded, poorly ventilated environments (12, 22–25), such as school classrooms (26, 27) and conference rooms (10) (Figure 1). Most humans are therefore chronically exposed to elevated *p*CO_2_ levels comparable to or higher than the highest *p*CO_2_ experienced in the Earth's atmosphere over the past 400 Myr (28), which is unprecedented since the appearance of mammals (Figure 1). Accordingly, the pH of human blood and tissues is likely shifted to values significantly below (<-0.1 pH units) the 7.35 pH optimum throughout significant spans of our lives.

The important role of elevated CO_2_, and the associated decrease in blood pH in affecting human respiration, e.g., Haldane effect (29), and reducing hemoglobin affinity for O_2_ with increasing CO_2_, i.e., Bohr effect (30), have been known for at least one century, which therefore leads to the expectation that the elevated CO_2_ humans now experience may affect human performance (1, 12, 25, 31). Lethal levels of CO_2_ are on the order of 10% in air (32), corresponding to a lethal threshold blood pH of about 6.8 (Figure 1), and the recommended occupational threshold level for 8-h time-weighted average exposures to CO_2_ is 5,000 ppm (33). Experimental assessments show that exposure of pigs to air highly enriched in CO_2_ leads to rapid brain pH decline, declining to lethal levels of 6.7 within 1 min (34). Hence, exposure to high CO_2_ concentration in ambient air is used to promote pre-slaughter anesthesia in swine (34).

Exposure to *p*CO_2_ levels > 600 ppm has been shown to lead to the “sick-building syndrome,” resulting in irritation, fatigue, anxiety, headaches, and poor cognitive performance (22, 24–26, 35) and sleep apnea (36), linked to elevated *p*CO_2_ in blood (10). Exposure to elevated CO_2_ also leads to the emotional responses of fear and panic in humans, a behavior that has also been experimentally confirmed in rats (37). Elevated indoor CO_2_ concentrations, well in excess of 1,000 ppm CO_2_, are also characteristic of farm animal houses [e.g., (38, 39)]. Yet, there is a surprising lack of assessments in the veterinary literature of the effects of chronic exposure to elevated CO_2_ levels consistent with indoor concentrations, resulting in potential effects on animal health being ignored in reviews on the effects of climate change on farm animals (40).

The lowest ambient CO_2_ values that have been tested in chronic exposure studies with animal models are 2,000 and 3,000 ppm, including experiments on dogs, rats, and guinea pigs, as well as observations on humans in military submarine patrols (23, 41). These studies show that chronic exposure to elevated CO_2_ leads to increased gastric-acid secretion, increased calcification in kidneys, and increased CO_2_ uptake, but reduced Ca concentration in bones across mammal species tested, including humans, rats, guinea-pigs, pigs, and dogs (23, 41).

Existing arguments on the effect of elevated CO_2_ on human health focus on the effects of exposure to acute CO_2_ levels and/or cognitive responses to elevated indoor CO_2_ levels (24, 25). We submit, however, that broader, more profound but subtle changes may operate through disruptions of human proteome functions under chronic exposure to elevated CO_2_ levels, provided the associated reduction in blood pH below the 7.35 optimum. Yet, the effects of human exposure to elevated CO_2_ levels have been mostly assessed in terms of physiological and cognitive levels (23). The effects of human exposure to elevated CO_2_ on the proteome, which can elicit broad systemic impacts, have been poorly explored.

Here, we put forth the hypothesis that chronic exposure to elevated *p*CO_2_ with increasing atmospheric CO_2_ and extended time spent in confined, crowded indoor atmospheres is a major, largely overlooked, driver of change in the performance of the human proteome. Specifically, we describe the multiple ways in which lowered pH resulting from elevated *p*CO_2_ levels may affect the performance of the human proteome, leading to metabolic changes favoring multiple health syndromes characterizing modern, urban societies.

## Discussion

A wealth of evidence for the effects of pH on the performance of proteins and, more broadly, biomolecules is available (Table 1). Unfortunately, many experiments assess pH ranges that are too broad (typically 2 pH units, Table 1), often using buffers (e.g., PBS—Phosphate-Buffered Saline) and acids (e.g., HCl—hydrochloric acid) to manipulate pH rather than the CO_2_-bicarbonate system operating in mammals. The 280 ppm *p*CO_2_ under which human proteins evolved, and that dictated the standard blood pH of 7.35 (Figure 1) at which the performance of most human proteins is optimized, is no longer experienced by humans, since atmospheric *p*CO_2_ has already exceeded 400 ppm and is expected to rise further (Figure 1). The shift in pH expected from the seven-fold increase in *p*CO_2_ experienced regularly by humans (from “standard” 280 ppm to the upper level of about 5,000 ppm in moderately crowded indoor environments, Figure 1), is calculated to involve a 0.3–0.4 pH unit reduction in blood (10, 42), cascading through all tissues and organs (Figure 1). Intracellular pH responds to changes in extracellular pH, with this coupling arising because the proteins that regulate intracellular pH are also sensitive to extracellular pH. This leads to a re-balancing of transmembrane acid–base fluxes that alter the steady-state intracellular pH, as recently demonstrated using high-throughput intracellular pH techniques (9). The rising *p*CO_2_ in the environment is, therefore, expected to lead to chronic acidosis, resulting in pH in human fluids chronically below the optimal pH of activity for the majority of human proteins (Table 1). Humans, therefore, regularly experience a dynamic pH range [[pH]*p*CO_2_] ranging from 7.1 to 7.45 (Figure 1), with the lower lethal pH threshold for humans being about 6.8 (43–45).

The pH-dependence of protein performance has been known for decades, but detailed understanding of the processes involved continues to grow (Table 1), providing evidence for a narrowly defined pH range for optimal performance of the human proteome [e.g., (46); Table 1, Figure 1], with the exception of proteins operating along the digestive tract, which perform at much lower pH values. Lowered pH affects protein folding, thereby affecting their ability to function properly (Table 1), through redistribution of surface charges that govern molecule-molecule interactions. For instance, when pH in the blood is reduced, the fraction of protonated histidine (His39) imidazole side chains in human serum albumin increases. This is important because human serum albumin is a major component of human blood. This affects the His39 role in preserving the required fraction of free thiols (-SH) in the blood, for which albumin (Cys34 residue existing in a reduced state) is the main provider [(47); Table 1]. pH changes within *p*CO_2_ also affect the aggregation of proteins, such as transthyretin in blood, synucleins and amyloid β-peptides in neuronal axons, and the human hormone amylin (Table 1), present in pancreas. Amylin misfolds into amyloid plaques with reduced pH, as a result of the protonation of the His18 residue that acts as an electrostatic switch to inhibit aggregation (48). Interactions between proteins and other proteins and/or nucleic acids are also likely to be pH dependent, affecting the formation of active complexes involved in gene regulation and repair mechanisms (Table 1). Changes in pH are likely to affect charge distribution on RNA-binding proteins (RBPs), thereby modulating protein-RNA interactions (Table 1). Moreover, RBPs are prone to misfolding as well as aggregation processes with shifting pH values (49, 50). Changes in the interactome with changes in pH also apply to their affinities for exogenous molecules such as drugs (51). Allosteric changes driven by molecular interactions are also pH dependent, as exemplified by the classic case of competition between O_2_ and CO_2_ binding sites in hemoglobin (52) (Figure 1).

Therefore, a reduction in pH with increased ambient *p*CO_2_ elicits a broad range of changes in the human proteome and the associated interactome (Table 1). Whereas these changes need to be better assessed within the relevant pH range (7.1–7.45) imposed by changes in CO_2_ (53), we submit that changes in the performance of the proteome with increasing *p*CO_2_ in the environment are very likely to contribute to a number of health syndromes affecting modern, urban societies (31). Brain performance is believed to be particularly sensitive to shifting pH (54). This is consistent with extensive epidemiological evidence that humans spending significant times in environments with elevated *p*CO_2_ suffer from attention deficit and learning disorders [(26, 55); e.g., Figure 1]. Moreover, the aggregation of proteins into insoluble amyloid fibrils, which could be achieved with a reduction in pH, is the hallmark of many highly debilitating brain pathologies such as Alzheimer's or Parkinson's disease.

The range of syndromes that may be rooted in chronic exposure to elevated *p*CO_2_ extend broadly to include diabetes (e.g., through pH-dependent changes in aggregation, Table 1), neurodegeneration (e.g., pH-dependent changes in synuclein and amyloid β-peptide aggregation, Table 1), schizophrenia and bipolar disorder (54), respiratory syndromes (e.g., pH-dependent changes in hemoglobin O_2_ affinity, i.e., the the Bohr effect, and α-trombin inhibitor activity, Table 1), osteoporosis (pH-dependent changes in carbonic anhydrase, Table 1), inflammation (23), and cancer (e.g., pH-dependent changes in GST, Table 1).

The role of elevated *p*CO_2_ is not limited, however, to indirect effects driven by reduced pH, but may also derive directly from the role of CO_2_ as a signaling molecule, broadly interpreted as corresponding to elevated metabolic demand. Exposure to elevated CO_2_ leading to a rise in arterial *p*CO_2_ and decrease in pH are detected by peripheral and central chemoreceptor cells and elicit a number of physiological and emotional responses. Emotional responses in humans exposed to elevated *p*CO_2_ result in distress behaviors, including fear, anxiety, and escape reactions. Similar responses have been confirmed experimentally on rat models exposed to very high CO_2_ levels (37), and involve the amygdala acting as a chemoreceptor for changes in *p*CO_2_/H^+^ and releasing adaptive responses. For instance, specific brainstem neurons (e.g., retrotrapezoid nucleus) are activated by elevated *p*CO_2_ and stimulate breathing (14, 36). The list of human cell types that functionally sense CO_2_, typically involving soluble adenylyl cyclase acting in tandem with carbonic anhydrase, is constantly expanding (56). Human preadipocytes were recently shown to sense elevated CO_2_, along the pH range of 7.1–7.4, triggering a cascade of responses that eventually lead to adipogenesis and obesity (53), a major problem of modern, sedentary societies exposed to chronically elevated *p*CO_2_ indoors. However, experiments testing such responses to chronic exposure at indoor (e.g., 2,000 and 3,000 ppm) CO_2_ levels are missing.

Consideration of the anomalous proteome of the naked mole rat (NMR, *Heterocephalus glaber*) provides further indirect evidence for the role of exposure to stable ambient CO_2_ on the evolution of a pH optimum for the performance of the mammalian proteome. NMR, a rodent from the African Horn, has received considerable attention because of their remarkable longevity and lack of cancer [over two decades compared to <3 years of other rodents; (57)]. NMR inhabits underground galleries under extreme crowding and, as a consequence, is adapted to extraordinarily high *p*CO_2_ levels in their burrows (58, 59). Remarkably, the only difference found thus far between NMR and its closest rodent relatives inhabiting outdoor environments is the substantially enhanced proteome fold stability, the resistance to reactive oxygen species (60), and/thus efficient DNA repair mechanisms in their acidized blood (60, 61). However, their remarkable properties vanish when NMRs are forced to live at atmospheres with ambient *p*CO_2_ (62). Thus, the adaptation of NMRs to life under high *p*CO_2_ levels is dependent, critically, on the adaptation toward increased protein stability at their low blood pH, suggesting their proteome has adapted to optimal performance at elevated *p*CO_2_ (therefore reduced pH) in their habitat. Hence, the proteome may evolve to perform at different optimal *p*CO_2_ conditions reflecting those prevailing in the habitat, but the *p*CO_2_ range for optimal performance remains narrow.

The link between elevated *p*CO_2_ and proteome stability and associated human health syndromes proposed here remains hypothetical and needs additional experimentation. This requires, however, that experiments with human cell lines be conducted under carefully controlled and monitored pH conditions (9). Unfortunately, most experiments using human cell lines neglect to control and monitor pH (9), and often use buffers absent from human fluids in the formulation of the culture medium (9). Equipment and standards to perform experiments with human cell lines controlling pH with CO_2_ additions (within the range 6.8–7.4), and to monitor pH and CO_2_ levels through the experiments are urgently needed. Protocols to examine the effects of pH changes within the range 6.8–7.4 on the structure and function of biomolecules are also required, as most assessments have been conducted, for technical convenience, across very large pH ranges of 2 pH units and greater (Table 1), which are beyond the physiological range for humans and mammals (Table 1).

In conclusion, we hypothesize that the five-fold rise in *p*CO_2_ levels that extant humans experience in their increasingly urban and sedentary lives relative to those they experienced during 1 million years of evolution may have systemic but yet overlooked impacts on the performance of the human proteome. Whereas, most research has focused on the consequences of acute exposure to extreme *p*CO_2_ levels, widespread impacts on the performance of the human proteome are also evident under the narrower pH range (7.10–7.45) corresponding to the chronically elevated CO_2_ levels most humans experience. Existing evidence suggests that these impacts are consistent with the diseases characteristic of modern, urban, and crowded lifestyles, such as diabetes, obesity, attention disorder, osteoporosis, cancer, anxiety, and prevalence of mental and respiratory disorders. The role of components of this lifestyle, such as unhealthy diets and sedentarism, on health has been extensively examined. However, the role of chronic exposure to elevated CO_2_ has only been addressed in relation to respiratory performance under acute exposure, while the more systemic effects operating through pH regulation of proteome performance have been largely overlooked. Yet, the link between elevated CO_2_ and modern lifestyle health syndromes proposed here provides a non-exclusive but parsimonious explanation for these disorders that has yet to be addressed. Ignoring the hypothesis formulated here may result in shifting health baselines of human populations that are continuously exposed to rising CO_2_ in the environment as humans adhere to increasingly crowded indoor, urban lifestyles. The suite of health syndromes associated with chronic exposure to elevated CO_2_ in indoor environments is not unavoidable (55), as solutions may be relatively simple. For instance, efficient ventilation systems in intelligent buildings combined with carbon capture technologies may be deployed in buildings to ensure CO_2_ levels indoors are conducive to healthier lifestyles (63, 64). Addressing the systemic effects that elevated CO_2_ may have on the human proteome requires the development of new approaches, standards, and equipment to examine its performance under narrow pH ranges. These efforts need progress beyond the very rough control of pH (typically 6.0–7.4) and the use of buffering systems absent from the human system, rather than the CO_2_-bicarbonate buffer, that characterize the majority of current research with human cell lines.

## Author Contributions

All authors listed have made a substantial, direct and intellectual contribution to the work, and approved it for publication.

## Conflict of Interest

The authors declare that the research was conducted in the absence of any commercial or financial relationships that could be construed as a potential conflict of interest.
