# Supplementary material for: Hypothesis: Potentially Systemic Impacts of Elevated CO2 on the Human Proteome and Health
Source: Front Public Health. 2020 Nov 16;8:543322. doi: 10.3389/fpubh.2020.543322 (PMC7701242; doi:10.3389/fpubh.2020.543322)
Supplement: Supplementary file 1 [file Data_Sheet_1.PDF]

Supplementary 1.

References cited in Table 1.

S1 M. Fasano *et al.*, The extraordinary ligand binding properties of human serum albumin. *IUBMB Life*. **57** (2005), pp. 787–796.

S2. S. Sankaranarayanan *et al.*, Serum albumin acts as a shuttle to enhance cholesterol efflux from cells. *J. Lipid Res.* **54**, 671–676 (2013).

S3. A. J. Stewart *et al.*, Role of Tyr84 in controlling the reactivity of Cys34 of human albumin. *FEBS J.* **272**, 353–362 (2005).

S4. C. W. P. Voet D. , Judith G., *Fundamentals of Biochemistry: Life at the Molecular Level* (2008).

S5. B. Ulmasov *et al.*, Purification and kinetic analysis of recombinant CA XII, a membrane carbonic anhydrase overexpressed in certain cancers. *Proc Natl Acad Sci U S A*. **97**, 14212–14217 (2000).

S6. D. Zappulla, in *Air Pollution: Sources, Prevention And Health Effects* (Nova Science Publisher, 2013).

S7. A. D. Kenny, Role of carbonic anhydrase in bone: Plasma acetazolamide concentrations associated with inhibition of bone loss. *Pharmacology*. **31**, 97–107 (1985).

S8. A. Hulikova, N. Aveyard, A. L. Harris, R. D. Vaughan-Jones, P. Swietach, Intracellular carbonic anhydrase activity sensitizes cancer cell pH signaling to dynamic changes in CO2partial pressure. *J. Biol. Chem.* **289**, 25418–25430 (2014).

S9. S. C. Frost, R. McKenna, *Carbonic Anhydrase: Mechanism, Regulation, Links to Disease, and Industrial Applications* (Springer, London, 2014).

S10. C. Ward *et al.*, Carbonic Anhydrase IX (CAIX), Cancer, and Radiation Responsiveness. *Metabolites*. **8**, 13 (2018).

S11. J. R. Casey, S. Grinstein, J. Orlowski, Sensors and regulators of intracellular pH. *Nat. Rev. Mol. Cell Biol.* **11**, 50–61 (2010).

S12. A. D. Balgi *et al.*, Regulation of mTORC1 signaling by pH. *PLoS One*. **6**, e21549 (2011).

S13. R. A. Saxton, D. M. Sabatini, mTOR Signaling in Growth, Metabolism, and Disease. *Cell*. **168** (2017), pp. 960–976.

S14. L. Jasso-Gutiérrez, A. Vargas-Origel, E. Puente-Bañuelos, G. Romero-Villaseñor, Effects of pH on alpha 1-antitrypsin activity in premature infants with idiopathic respiratory distress syndrome. *Arch. Invest. Med. (Mex).* **10**, 7–14 (1979).

S15. I. Haq *et al.*, Deficiency mutations of alpha-1 antitrypsin: Effects on folding, function, and polymerization. *Am. J. Respir. Cell Mol. Biol.* **54**, 71–80 (2016).

S16. C. B. Glaser, L. Karic, A. B. Cohen, Low pH stability of alpha-1-antitrypsin. *BBA - Protein Struct.* **491**, 325–330 (1977).

S17. S. Urien, F. Brée, B. Testa, J. P. Tillement, pH-dependency of basic ligand binding to alpha 1-acid glycoprotein (orosomucoid). *Biochem. J.* **280 (Pt 1**, 277–80 (1991).

S18. T. Miura, A. Hori-I, H. Mototani, H. Takeuchi, Raman spectroscopic study on the copper(II) binding mode of prion octapeptide and its pH dependence. *Biochemistry*. **38**, 11560–11569 (1999).

S19. R. M. Vernon *et al.*, Pi-Pi contacts are an overlooked protein feature relevant to phase separation. *Elife*. **7** (2018), doi:10.7554/eLife.31486.

S20. L. J. Colwell, M. P. Brenner, K. Ribbeck, Charge as a selection criterion for translocation through the nuclear pore complex. *PLoS Comput. Biol.* **6**, e1000747 (2010).

S21. J. A. Riback *et al.*, Stress-Triggered Phase Separation Is an Adaptive, Evolutionarily Tuned Response. *Cell*. **168**, 1028–1040.e19 (2017).

S22. E. Gomes, J. Shorter, The molecular language of membraneless organelles. *J. Biol. Chem.* (2018), doi:10.1074/jbc.TM118.001192.

S23. J. Sheu-Gruttadauria, I. J. MacRae, Phase Transitions in the Assembly and Function of Human miRISC. *Cell*. **173**, 946–957.e16 (2018).

S24. A. Aguzzi, M. Altmeyer, Phase Separation: Linking Cellular Compartmentalization to Disease. *Trends Cell Biol.* **26**, 547–558 (2016).

S25. Y. Wang, A. Lomakin, R. F. Latypov, G. B. Benedek, Phase separation in solutions of monoclonal antibodies and the effect of human serum albumin. *Proc. Natl. Acad. Sci. U. S. A.* **108**, 16606–11 (2011).

S26. G. Maulucci *et al.*, Phase separation of the plasma membrane in human red blood cells as a potential tool for diagnosis and progression monitoring of type 1 diabetes mellitus (2017), doi:10.1371/journal.pone.0184109.

S27. N. Bley *et al.*, Stress granules are dispensable for mRNA stabilization during cellular stress. *Nucleic Acids Res.* **43**, e26–e26 (2015).

S28. A. F. Harrison, J. Shorter, RNA-binding proteins with prion-like domains in health and disease. *Biochem. J.* **474**, 1417–1438 (2017).

S29. S. Jha *et al.*, PH dependence of amylin fibrillization. *Biochemistry*. **53**, 300–310 (2014).

S30. G. M. Moriarty *et al.*, A pH-dependent switch promotes β-synuclein fibril formation via glutamate residues. *J. Biol. Chem.* **292**, 16368–16379 (2017).

S31. P. E. Fraser, J. T. Nguyen, W. K. Surewicz, D. A. Kirschner, pH-dependent structural transitions of Alzheimer amyloid peptides. *Biophys. J.* **60**, 1190–1201 (1991).

S32. S. Skoulakis, J. M. Goodfellow, The pH-dependent stability of wild-type and mutant transthyretin oligomers. *Biophys. J.* **84**, 2795–2804 (2003).

S33. K. Satoh, The high non-enzymatic conjugation rates of some glutathione s-transferase (gst) substrates at high glutathione concentrations. *Carcinogenesis*. **16**, 869–874 (1995).

S34. G. Litwack, B. Ketterer, I. M. Arias, Ligandin: A hepatic protein which binds steroids, bilirubin, carcinogens and a number of exogenous organic anions. *Nature*. **234** (1971), pp. 466–467.
